# A Robust Angular Rate Sensor Utilizing 2:1 Auto-Parametric Resonance Excitation

**DOI:** 10.3390/s22207889

**Published:** 2022-10-17

**Authors:** Bhargav Gadhavi, Farid Golnaraghi, Behraad Bahreyni

**Affiliations:** School of Mechatronic Systems Engineering, Simon Fraser University, Surrey, BC V3T 0A3, Canada

**Keywords:** Microsensors, angular rate sensor, nonlinear sensing, auto-parametric resonance

## Abstract

This paper presents a single-axis angular rate sensor that is robust to variations in its operating voltage and frequencies. The sensor is developed to overcome the shortcomings of conventional mode-matched Micromachined Vibratory Gyroscopes in open loop operations, namely narrow frequency bandwidths and unstable scale factors. The developed sensor utilizes inherent forcing and inertial nonlinearities from electrostatic forces and fabrication imperfections to auto-parametrically excite the sense mode via 2:1 auto-parametric resonance, which yields a broader bandwidth frequency response for the sensor’s sense mode. The experimental results demonstrated −3 dB frequency bandwidth of 500 Hz, a scale factor of 50 μV/°/s, and a dynamic range of ±330°/s.

## 1. Introduction

Micromachined Vibratory Gyroscopes (MVGs) [1] or angular rate sensors are used in various motion-sensing applications, including automotive stability control [2], image stabilization in cameras, and inertial measurement units (IMUs) of wearable devices [3,4]. They are often designed as mode-matched sensors to maximize the scale factor sensitivity of the MVG’s output [1] by using identical drive and sense mode resonant frequencies. However, perfect mode matching is difficult to achieve due to fabrication imperfections and fluctuations in operating conditions, such as variations in actuation voltage. Further, MVGs are designed to gain high-quality factors, or Q factors, in drive and sense modes to achieve higher scale factors and resolution of the sensor, yielding the modal resonance responses with narrow frequency bandwidths. Although a high Q factor amplifies the sensor’s output dramatically, it also leaves its output vulnerable to variations in either operating frequency or the sense mode resonant frequency. However, if a broader frequency response for the sense mode can be obtained, then scale factor sensitivity to those variations would decrease. This paper demonstrates the utility of the nonlinear resonance phenomenon known as the 2:1 auto-parametric resonance (AR) in broadening the designed sensor’s frequency response for its sense mode.

**Figure 1:**
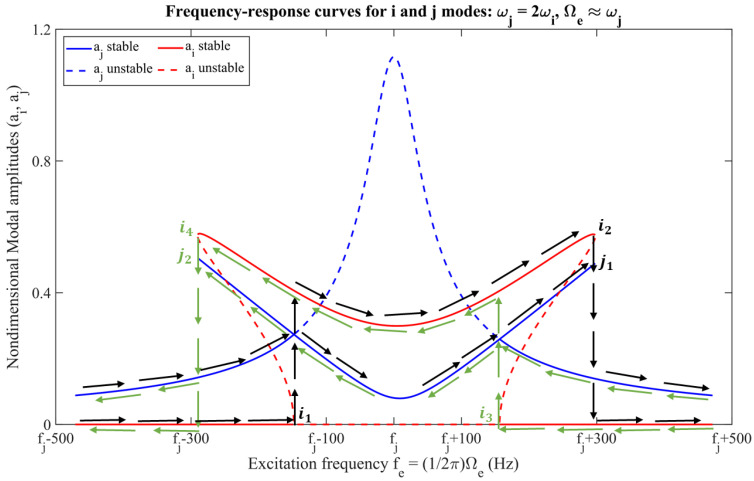
Typical frequency response of a 2 DOF, nonlinearly coupled oscillator for the primary resonance of mode j at 2:1 AR.

Recent studies [5,6,7,8,9] have revealed that for an oscillatory system, such as an MVG, if two or more of its linear resonant frequencies are commensurate, those corresponding modes could be strongly coupled depending on the order of nonlinearities in the system. Notably, for a multi-degree of freedom system with quadratic nonlinearities, a linear natural frequencies’ commensurability of $\omega_{j}\approx 2\omega_{i}$ for any of the system’s two modes i and j, could cause a special kind of resonance, known as a 2:1 internal resonance or 2:1 AR [10] between those two modes. For such a nonlinearly coupled system, when its higher frequency mode, mode j, is externally excited via a harmonic force, such as $F_{e}=F_{0}\sin\left(\Omega_{e}t\right)$, at its primary resonance, $\Omega_{e}\approx \omega_{j}$, and the forcing amplitude is greater than some threshold amplitude $\left(F_{0}>F_{0}^{*}\right)$ then due to quadratic coupled nonlinearities, mode i gets internally or auto-parametrically excited via 2:1 AR, as shown in Figure 1. The black and green arrows in the figure represent the direction of the frequency sweep. The frequency bandwidth of the mode i, due to 2:1 AR, is both considerably wider and flatter compared to the conventional linear resonance response. Figure 1 also demonstrates the nonlinear jump phenomenon and readers are encouraged to refer to the references [5,6] for detailed behavior of 2:1 AR.

There are many sources of nonlinearities in the micro-systems. Examples of sources of nonlinearity in MEMS include material nonlinearities in piezoelectric materials [11], geometric nonlinearities due to micro-cantilevers experiencing large deformations [8,9], addition of coupled motion in the given mode of oscillation or non-conformity leading to irregular mass distribution [12], damping nonlinearity from inherently nonlinear squeeze film damping in MEMS [13], and forcing nonlinearities from inherently nonlinear electrostatic forces in parallel plate electrodes [14,15,16]. MVGs comprise multiple-beam springs and actuation drives and, hence, are susceptible to inertial, damping and forcing nonlinearities among the aforementioned sources.

Due to modal interactions through AR, the nonlinearities and commensurable linear resonant frequencies in MEMS resonators may cause the resonator response in corresponding modes to be strongly coupled and dramatically amplified yet bounded by the damping and nonlinearities present in the system. Different cases of AR, including 1:1, 1:2, 2:1, 1:3, and 3:1 based on electrostatic actuation have been demonstrated in various studies of MEMS resonators [14,15,16,17,18,19,20,21,22,23,24,25,26,27,28]. We present some of these relevant AR studies in MEMS resonators and the challenges to successfully implementing AR.

A study by Daqaq et al. [14] focused on nonlinear modal interactions, due to both 1:2 and 2:1 AR, in a micromirror. They modelled the micromirror with two torsion and bending nonlinearly-coupled modes via electrostatic forces using the lumped mass parameter model. They found that upon electrostatically exciting the mirror’s lower frequency mode—its torsional mode, with a range of DC bias voltage above some threshold voltage, the non-externally excited higher frequency mode, bending, is auto-parametrically excited via 1:2 AR. They concluded that accidentally operating the micro scanner at the critical DC voltage while resonating the torsional mode could cause the scanner’s movements in the bending mode. While this study intended to avoid 2:1 AR, the following studies focus on utilizing 2:1 AR.

Vyas et al. [15,16] analyzed, both analytically and experimentally, a T-beam micro-resonator with a frequency ratio of close to 2:1 between the higher, in-plane flexural mode and the lower, out-of-plane torsional mode. They demonstrated that when the flexural mode is excited at its resonance by electrostatic forces with its AC voltage amplitude greater than a threshold voltage of 5 mV, the torsional mode gets auto-parametrically excited due to 2:1 AR. Their study demonstrated that 2:1 AR could amplify the response of indirectly excited mode, and wider frequency bandwidths can be obtained to enhance the resonator’s sensing performance. In the studies by Sarrafan [18] and Noori [19], electrostatically-actuated and capacitively-sensed MEMS resonators based on the 2:1 AR were developed, and experimental tests demonstrated frequency response curves for the auto-parametrically excited mode with wider frequency bandwidths. Sarrafan [18] proposed an H-shaped, 2:1 AR-based MEMS resonator and tested it for angular rate sensing applications. Although the −3 dB bandwidth values for the sensor’s sense mode frequency response are not reported, the overall bandwidth of the sense mode response at 2:1 AR appears to be about 3.5 kHz, which is a remarkably broader bandwidth response. They did not conduct in-depth testing to evaluate all gyroscope performance parameters for their sensor, but they tested its scale factor and full-scale range to be 11 μV/°/s and ±200°/s, respectively. Kumar et al. [22] studied, analytically and through the Finite Element Method (FEM), all possible AR cases for the first three modes of a micro-resonator consisting of a clamped-clamped beam. Their unique finding was that out of all potential AR cases for the first three modes of the beam, only 2:1 AR between the third and the first modes can exhibit modal coupling that allows the transfer of energy between those two modes.

On the other hand, studies in [25,27,28,29] demonstrated how AR could be utilized in designing self-sustaining MEMS oscillators with nonlinearly coupled modes to output the desired reference frequency with fewer fluctuations. In [25], a fixed-fixed microbeam capable of resonating at 2:1 AR was experimentally tested using a closed loop system, and frequency stability of 13 ppm was achieved for the designed MEMS oscillator. These studies indicate AR-based MEMS resonators’ potential to achieve very low-frequency noise performance while operating them in the nonlinear regime. In recent times, AR is has also been utilized in a mass sensor for sensitivity enhancements of the sensor [30], in energy harvesters for achieving wider frequency bandwidths than linearly resonating harvesters [20,31,32], and in atomic force microscopy for Young’s modulus sensitivity enhancements [21]. Gobat et al. [33] proposed a nonlinear Model Order Reduction technique to convert the high-fidelity finite element models to a model of a few degrees of freedom only. They validated their proposed numerical tool through analytical and experimental investigation of 1:2 internal resonance in a tuning fork MEMS gyroscope. Marconi et al. [34] demonstrated the exploitation of nonlinearities in designing frequency-matched MEMS gyroscopes. They showed that the sensor’s angular rate sensitivity could be boosted by sweeping the drive amplitude, which in turn causes the drive and sense mode frequencies to match up.

The test and characterization procedures to obtain MVG’s performance measures have been well developed and can be found in IEEE standard 1431 [35] and other literature [36,37]. Amplitude Modulated (AM) MVGs could be broadly classified as either mode-matched MVGs or mode-split MVGs. The former type employs the necessary design and operational strategies to match the drive and sense mode’s resonant frequencies to maximize the sensor’s angular rate sensitivity and noise performance at the expense of a narrow frequency bandwidth for the sense mode. On the other hand, few manufacturers and researchers developed robust MVGs of the second type by splitting the drive and sense mode resonance frequencies to detect angular rates in a wider frequency spectrum but via sacrificing gain or scale factor of the sensor. Table 1 summarizes performance parameters of state-of-the-art MVGs with the last three rows of commercial single-axis MVGs. We first review mode-matched MVGs and different closed-loop strategies that could mitigate their bandwidth problems.

Ideally, mode-matched MVG’s drive and sense modes should be decoupled in the absence of angular rate; however, fabrication imperfections cause additional elastic and damping couplings that lead to quadrature error of the sensor. Zaman [38] designed a z-axis (yaw rate) mode-matched tuning fork MVG with dedicated quadrature compensation electrodes that apply torque to proof masses to reduce their fabrication-induced misalignment, nulling the quadrature error. Their sensor demonstrated a scale factor of 88 mV/°/s with other performance parameters listed in Table 1. However, without an auto-tuning of some kind for mode-matching, the sensor becomes vulnerable to losing its angular rate. In a real-life application, a mismatch of >0.5 Hz could be caused by a high-frequency input rate or environmental temperature fluctuations. Further, due to narrower bandwidth, the sensor also experiences a limited full-scale range of ±50°/s.

Liu et al. [39] developed a single-axis mode-matched MVG with a doubly decoupled structural design that encompasses three resonating masses to further decouple the drive and sense modes from each other. Their sensor’s circuit included a close loop drive mode operation, and the readout mode demonstrated better full-scale range and control over scale factor nonlinearity, as shown in Table 1. Wang et al. [40] developed a mode-matched SOI-based tuning fork MVG with multiple beams to achieve high Q factors, 255.55 k and 103.39 k for the drive and sense modes, respectively. Their sensor was tested with a closed-loop drive and an open-loop sense mode, and its performance parameters are listed in Table 1. Sharma et al. [41] developed a CMOS application-specific integrated circuit (ASIC) to control the bandwidth (1–10 Hz) of their MVG electronically. They proposed an electronic sensor control that allows the user to select either a wider frequency bandwidth (10 Hz) operation or a higher scale factor (88 mV/°/s) operation. Sonmezoglu et al. [42] developed an automatic mode matching closed-loop system for their fully decoupled MVG. Their sensor showed tactical-grade level noise performance and broader sensor bandwidth as compared to other mode-matched MVGs. The mechanical bandwidth of their sensor was only 8 Hz, but they were able to develop the system bandwidth of 42 Hz via a low pass filter for their sensor. A group of researchers at Robert Bosch published a mode-matched 3-axis MVG fabricated via a 20 μm structural poly-Si layer [43]. They employed closed-loop electronics to achieve low noise and offset drift performance of the sensor by suppressing electromechanical quadrature, and their measurements demonstrated bandwidth of 80 Hz. Jia et al. [44] developed a z-axis, automatically tuning mode matched MVG that utilizes a frequency tuning method based on a quadrature modulation signal. Their sensor showed a great degree of control over the scale factor linearity error of 28 ppm.

Aaltonen et al. [45] designed a 2-axis (x, y) low power (2.2 mA) compact MVG with drive and sense mode frequencies split. They developed a high aspect ratio sensing element with capacitive actuation and capacitive pseudo-continuous time readout to reduce the chip area. The performance parameters of their sensor’s x-axis sensing element are listed in Table 1. They achieved a wider bandwidth of 160 Hz without significantly decreasing noise performance. Wu et al. [46] developed a decoupled structure for their MVG on a 30 μm SOI substrate. Their sensor demonstrated the angular rate output to be above the drop of −3 dB in its amplitude for angular rate inputs up to 120 Hz, thus widening the sensor’s bandwidth. They employed a high resolution 16 bits analog to digital converter (ADC) to digitize the sensor’s output and measured a scale factor of 47.3 LSB/°/s with a considerably better full-scale range of ±500°/s. Similarly, the MVGs reported in [47,48] also show wider frequency bandwidths than any mode-matched MVGs listed in Table 1.

To summarize, mode-matched MVGs improve the sensor’s scale factor and noise performance at the cost of sensor bandwidth, which could still be marginally widened via either lowpass filters through external passive components or closed-loop control. However, to gain even wider sensor bandwidth and broader sensor’s full-scale range, mode split designs should be considered, which come at the expense of worsened scale factor and noise performance. On the other hand, AR-based sensing for an MVG in [17,18] has shown the potential of obtaining unusually wider sense mode frequency bandwidths which, according to the study in [27], could achieve higher stability of sense mode amplitudes and thus better noise performance. This paper presents a Robust Angular Rate Sensor (RARS) architecture encompassing AR-based sensing for angular rate measurements. Demonstrating a noise performance comparable to mode-matched MVGs, the sensor offers wider frequency bandwidth that results in simpler control electronics at the expense of a modest loss of scale factor.

## 2. Sensor Design and Fabrication

The sensor architecture of RARS is intended to employ all the requirements for achieving 2:1 AR in the sensor design. These requirements are: (i) the two orthogonal modes of interest must be nonlinearly coupled, (ii) natural frequency commensurability between the modes must be $\omega_{2}\approx 2\omega_{1}$, and (iii) the forcing amplitude of the directly excited drive mode should be higher than some threshold to cause an energy transfer to the auto-parametrically excited sense mode [5]. The first requirement, in this case, is met because of three types of nonlinearities, i.e., inertial nonlinearities arising from the microfabrication imperfections, the damping nonlinearities via nonlinear squeeze film damping, and excitation nonlinearities because of inherently nonlinear electrostatic forces. The required frequency commensurability is ensured by intentionally designing the modes of interest with $\omega_{2}\approx 2\omega_{1}$ and the needed threshold electrostatic force amplitude is achieved via a smaller gap of 1.75 μm between the resonating mass and steady electrodes. It should be noted that this frequency commensurability is also fine-tuned through electrostatic softening by adjusting the applied DC bias voltage to the device. Further, each fabricated sensor chip includes nine instances of the RARS with varying beam dimensions to yield the frequency ratio, $R_{f}=\omega_{2}/\omega_{1}$, in the range of [1.98 2.02] through finite element simulations. This design strategy ensured multiple instances of the sensor that demonstrated 2:1 AR at different DC bias voltage threshold values. Sensor instances with $R_{f}=2.04$ also demonstrated 2:1 AR by adjusting the applied DC bias voltage. Unlike mode-matched MVGs which require precise control for matching modal frequencies of drive and sense modes, the proposed strategy in this paper does not require such precise control and, thus, offers robustness to both fabrication imperfections and operating voltages.

**Figure 2:**
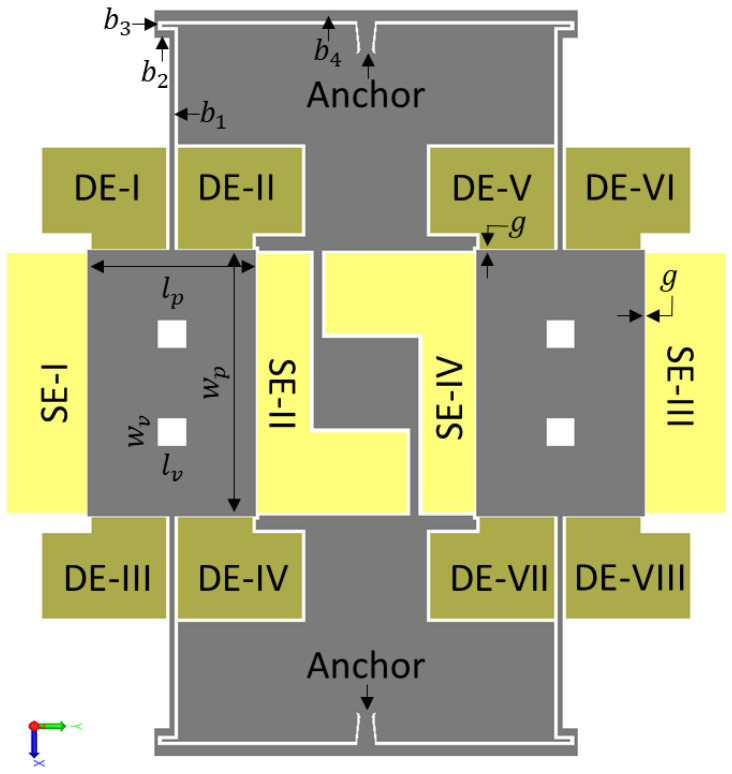
CAD model of RARS.

The CAD model of the sensor is shown in Figure 2, and the labelled dimensions are given in Table 2. Two identical rectangular proof masses are modelled as two tines of a tuning fork, connected to a common anchor in the center via four identical fishhook flexure systems. The fishhook design is employed to ensure the flexure system is compliant in the desired resonating modes of the proof mass and yet offers high stiffness in undesirable modes. The design utilizes two in-plane orthogonal modes, in X and Y directions, for its operation, as shown in the figure. RARS utilizes its in-plane X mode as sense mode and its in-plane Y mode as drive mode. Each proof mass plate is surrounded by six (two sense and four drive) stationary electrodes. For the anti-phase drive operation of RARS, the AC excitation voltage is applied to two pairs of drive electrodes, DE-I & DE-II (shorted) and DE-VII & DE-VIII (shorted). Further, as shown in Figure 2, every electrode is isolated from other electrodes by the silicon in a grey colour to reduce the interference from other electrodes and the sensor’s cross-axis sensitivity.

The actuation mechanism chosen for RARS is electrostatic because of two reasons, (i) a smaller gap, as variable gap actuation operation generates a higher electrostatic force required for 2:1 AR, and (ii) its ease of implementation. Variable gap actuation is employed rather than variable area actuation because the former actuation provides the more significant force per unit area required in RARS to achieve energy transfer from the drive mode to the sense mode via 2:1 AR. However, the pull-in behavior associated with the variable gap actuation may cause a snapping of the proof mass on an electrode. Pull-in voltages are calculated and simulated using finite element analysis (FEA) to avoid this. For similar reasons, variable gap capacitive detection is utilized for sensing operations of the RARS.

The dimensions annotated in Figure 2 are listed in Table 2. In the table, $b_{n}$ represents the surface area, $b_{n}=l_{n}\times w_{n}$, of beam n. The square vents on either proof mass are $40\times 40{\;\mu m}^{2}$ in size.

**Figure 3:**
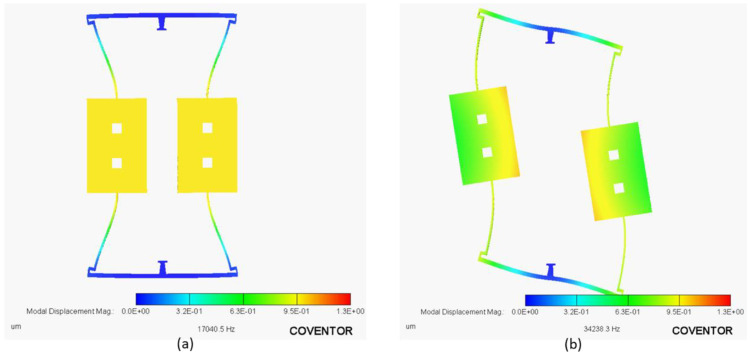
Modal analysis of RARS using CoventorMP: (**a**) Sense mode, (**b**) Drive mode.

A modal analysis using the FEA module of CoventorMP^®^ is conducted on the meshed model of RARS, and the results are shown in Figure 3. The simulated sense and drive mode natural frequencies are $f_{s}=17.04\;\mathrm{kHz}$ and $f_{d}=34.238\;\mathrm{kHz}$, respectively, with the frequency ratio of $R_{f}=2.009$. For calculating the spring constant of one fishhook flexure system, the following formulae, as given in [49,50], are utilized.

(1) $$K_{x}=\frac{2EI\left(2l_{t}l_{b}-l_{y}^{2}\right)}{2l_{b}l_{c}l_{t}-l_{c}l_{y}^{2}+2l_{a}l_{x}l_{y}-2l_{t}l_{a}^{2}-l_{b}l_{x}^{2}}\;K_{y}=\frac{2EI\left(2l_{t}l_{c}-l_{x}^{2}\right)}{2l_{b}l_{c}l_{t}-l_{c}l_{y}^{2}+2l_{a}l_{x}l_{y}-2l_{t}l_{a}^{2}-l_{b}l_{x}^{2}}$$

where

(2) $$I=\frac{w_{p}t_{p}^{3}}{12}\;l_{a}=-l_{1}l_{2}^{2}-2l_{1}l_{2}l_{3}+l_{2}l_{3}^{2}-2l_{1}l_{2}l_{4}-l_{1}l_{4}^{2}+2l_{2}l_{3}l_{4}+l_{3}l_{4}^{2}\;l_{b}=\frac{2}{3}l_{1}^{3}+2l_{2}l_{1}^{2}+2l_{3}l_{1}^{2}+\frac{2}{3}l_{3}^{3}-2l_{1}l_{3}^{2}+2l_{4}l_{1}^{2}+2l_{4}l_{3}^{2}-4l_{1}l_{3}l_{4}\;l_{c}=\frac{2}{3}l_{2}^{3}+2l_{3}l_{2}^{2}+2l_{4}l_{2}^{2}+\frac{2}{3}l_{4}^{3}+2l_{2}l_{4}^{2}\;l_{t}=l_{1}+l_{2}+l_{3}+l_{4}\;l_{x}=l_{2}^{2}+2l_{2}l_{3}+2l_{2}l_{4}+l_{4}^{2}\;l_{y}=-l_{1}^{2}-2l_{1}l_{2}-2l_{1}l_{3}-2l_{1}l_{4}-2l_{3}l_{4}+l_{3}^{2}$$

Here $l_{i}$ represents the length of the beam i=1…4. The calculated natural frequencies, by substituting the values from Table 2 in fishhook flexure stiffness equations, Equations (1) and (2), of the sense and drive modes are 17.124 kHz and 33.991 kHz, respectively, which are close to the natural frequencies found from modal analysis. Further, the pull-in voltage of the sense mode is calculated from [51], $V_{PI,x}=\sqrt{\frac{8K_{x}g^{3}}{27\varepsilon_{0}t_{p}w_{p}}}=68.12\;V$. Here, $K_{x}$ is the stiffness in *x* direction, and $\varepsilon_{0}$ is the vacuum permittivity, and its value is 8.85 pF/m.

To achieve the higher sensitivity of the proof mass displacements in the sense mode to applied angular rate inputs, the drive and sense mode’s quality factors, or shortly Q factors, must be high. Since vacuum packaging of the sensor allows low damping and hence high and stable Q factors over the sensor’s lifetime, the RARS devices are fabricated using the MEMS Integrated Design for Inertial Sensors (MIDIS) fabrication process of Teledyne DALSA. MIDIS offers a high vacuum with a minimum feature size of 1.5 μm, allowing us to design the gap between the static electrode and the moving proof mass to be in the range of 1.75 μm to 2 μm. The smaller gap in RARS causes higher electrostatic forces for the same applied voltage, which would actuate 2:1 AR.

**Figure 4:**
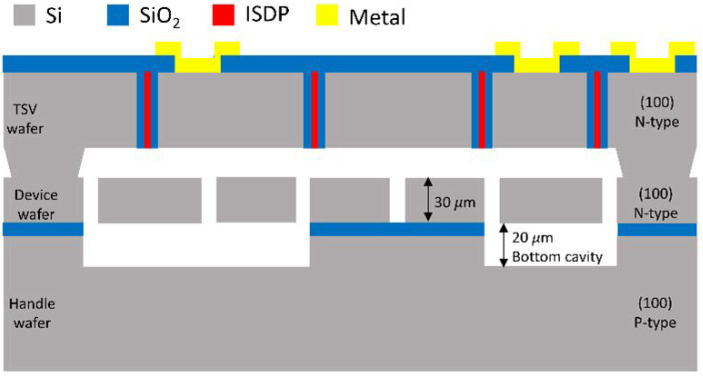
Sectional view of a microdevice made in MIDIS fabrication process [53].

The MIDIS process In Figure 4 is a wafer-scale vacuum encapsulation technology with an inner pressure of 1.5 Pa or 11.2 mTorr [52]. The device layer of RARS, including the resonator and its surrounding electrodes, is on a 30 μm thick N-type doped silicon (100) wafer, referred to as the device wafer. A P-type doped silicon (100) wafer, referred to as the handle wafer, acts as the substrate and creates the device wafer’s bottom cavity. The third wafer is N-type doped silicon (100) wafer, known as TSV (through silicon via) wafer, which creates the top cavity and TSV for the device layer. The vacuum is created by fusion bonding the TSV wafer and the handle wafer on either side of the device wafer. The TSVs in Figure 4 include ${\mathrm{SiO}}_{2}$ liners as insulators and in-situ doped polysilicon (ISDP) as conductors.

**Figure 5:**
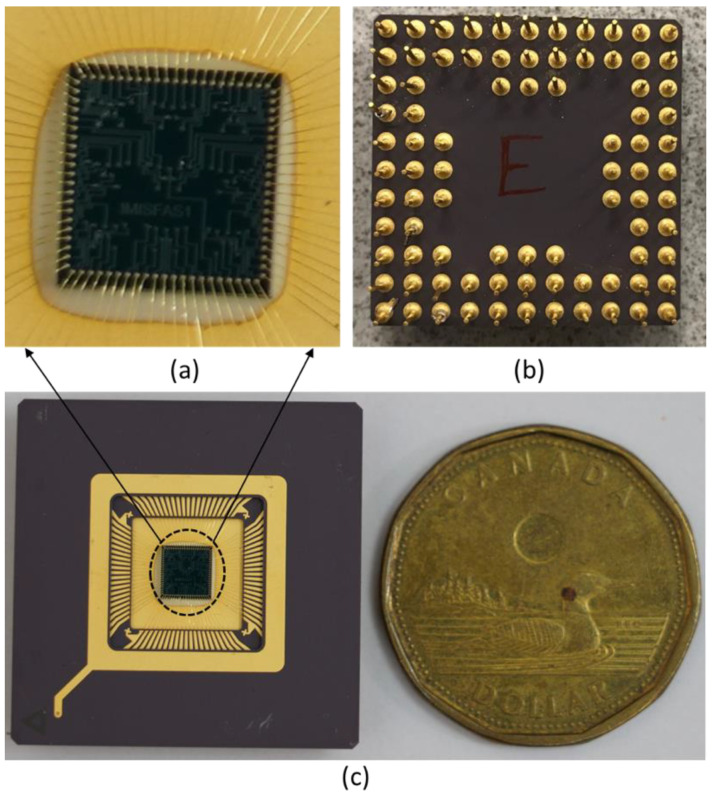
RARS fabricated device: (**a**) Zoom view of the top layer and wire bonding, (**b**) Top view of the package with 84 pins, (**c**) Bottom view of the package without a lid is compared to the size of a Canadian one-dollar coin.

Figure 5 shows the pictures of the RARS fabricated package. The vacuum-sealed chip is observed on the bottom of the package with its lid removed, as shown in Figure 5c. Its zoomed view, along with the wire bonding, is shown in Figure 5a. Notice that the 84 connections to the pads that are visible in Figure 5a correspond to the total of nine instances of the RARS on one vacuum encapsulated sensor chip. The top view of the package with 84 pins is shown in Figure 5b. Notice that these pictures were taken from the chip that served its purpose and was no longer required for further experiments.

## 3. Modeling of Sensor Dynamics

This section describes an intuitive understanding of how the RARS achieves angular rate sensitivity via nonlinear 2:1 AR by explaining its motion equations.

**Figure 6:**
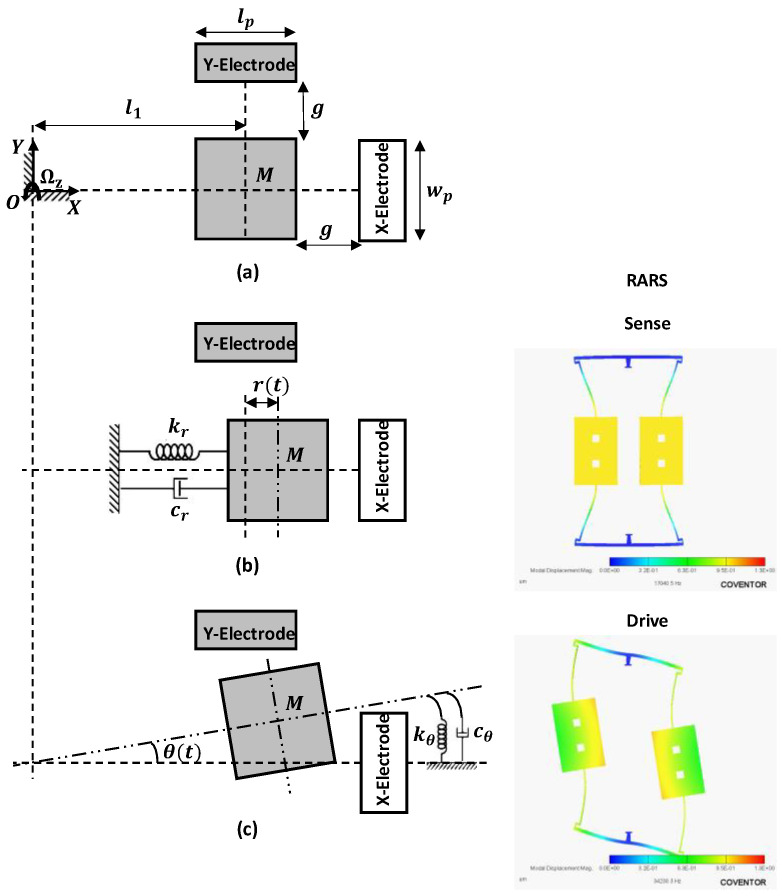
Lumped parameter model of RARS dynamics. (**a**) Undisplaced proof mass, (**b**) Proof mass displaced by r(t) in sense mode, (**c**) Proof mass displaced by θ(t) in drive mode.

Figure 6 shows a simplified two degrees of freedom, in-plane translation and torsional, lumped mass model of the RARS dynamics. For modelling the dynamics of both systems, the following assumptions are employed. (i) The left and right side resonators, proof mass–beam systems, are decoupled from each other through anchors. (ii) Since the sensor architectures are symmetric about the vertical axis connecting two anchors, studying only the left-side resonator dynamics is sufficient. (iii) Since the displacements of only the proof mass are utilized to both drive and sense the RARS, the proof mass, M, is assumed to be a rigid body that is anchored to the substrate via multiple mass-less flexible beams and anchors. The beams are modeled as mass-less springs and energy dissipation is assumed viscous and modeled as mass-less dampers. (iv) The system has two independent coordinates, r(t) for in-plane horizontal translation mode, sense mode, and θ(t) for in-plane torsional mode, drive mode. (v) The sensor is subjected to a constant angular rate, $\Omega_{z}$, with respect to the Z axis.

In Figure 6a, M represents the left side resonator’s proof mass of RARS. It is surrounded by two parallel plate capacitive electrodes, X-Electrode and Y-Electrode, in X and Y directions respectively, with a gap of g between each electrode and the proof mass. The length and width of the proof mass are $l_{p}$ and $w_{p}$ respectively. The fixed reference frame, OXYZ, has unit vectors $\hat{i}$, $\hat{j}$ and $\hat{k}$ in X, Y and Z axes, respectively. Further, $l_{1}$ is the radius of rotation of M from the origin of OXYZ frame.

Figure 6b shows the translation mode’s displacement, r(t), of M in X direction, its equivalent modal stiffness, $k_{r}$, and damping coefficient, $c_{r}$ of all the beams connected to the proof mass, M. Similarly, Figure 6c shows the in-plane torsional mode’s angular displacement, θ(t), of M from the previously displaced position and $k_{\theta }$ and $c_{\theta }$ represent its equivalent torsional stiffness and damping respectively.

The detailed derivation of the sensor’s motion equations and their numerical simulations is outside the scope of this paper. Using an energy approach, Lagrange’s equations are employed to derive the system’s motion equations as follows.

(3) $$\begin{array}{c} Ml_{1}{}^{2}\ddot{\theta }+C_{\theta }\dot{\theta }+K_{\theta }\theta =\underset{\underset{\mathrm{Linearly}\;\mathrm{coupled}\;\mathrm{Coriolis}}{⏟}}{\left[-2Ml_{1}\Omega_{z}\dot{r}\right]}-\underset{\mathrm{Quadratic}\;\mathrm{coupled}\;\mathrm{terms}}{\underbrace{\left[2Ml_{1}r\ddot{\theta }+2M\Omega_{z}r\dot{r}+2Ml_{1}\dot{r}\dot{\theta }\right]}}\\ -\underset{\mathrm{Cubic}\;\mathrm{coupled}\;\mathrm{terms}}{\underbrace{\left[Mr^{2}\ddot{\theta }+2Mr\dot{r}\dot{\theta }\right]}}+\underset{\mathrm{Electrostatic}\;\mathrm{force}}{\underbrace{\left[V_{YE}^{2}\left(\eta_{1}+\eta_{2}\theta +\eta_{3}\theta^{2}\right)\right]}} \end{array}$$

(4) $$\begin{array}{c} M\ddot{r}+C_{r}\dot{r}+K_{r}r=\underset{\mathrm{Constant}\;\mathrm{Centrifugal}}{\underbrace{\left[Ml_{1}\Omega_{z}{}^{2}\right]}}+\underset{\mathrm{Linear}\;\mathrm{terms}}{\underbrace{\left[\underset{\mathrm{Coupled}\;\mathrm{Coriolis}}{\underbrace{2Ml_{1}\Omega_{z}\dot{\theta }}}+\underset{\mathrm{Decoupled}\;\mathrm{Centrifugal}}{\underbrace{M\Omega_{z}{}^{2}r}}\right]}}\\ +\underset{\mathrm{Quadratic}\;\mathrm{coupled}\;\mathrm{terms}}{\underbrace{\left[Ml_{1}{\dot{\theta }}^{2}+2M\Omega_{z}r\dot{\theta }\right]}}+\underset{\mathrm{Cubic}\;\mathrm{coupled}\;\mathrm{terms}}{\underbrace{\left[Mr{\dot{\theta }}^{2}\right]}}+\underset{\mathrm{Electrostatic}\;\mathrm{force}}{\underbrace{\left[V_{XE}^{2}\left(\alpha_{1}+\alpha_{2}r+\alpha_{3}r^{2}\right)\right]}} \end{array}$$

where $V_{YE}$ and $V_{XE}$ are applied voltages to the Y and X electrodes, respectively. $C_{YE}$ and $C_{XE}$ are variable gap capacitances between M and Y electrode in θ mode and between M and X electrode in r mode, respectively. Following Figure 6, we can write $C_{XE}$ and $C_{YE}$ as follows.

(5) $$\begin{array}{c} C_{XE}=\frac{\varepsilon_{0}t_{p}w_{p}}{g-r}\\ C_{YE}=\frac{\varepsilon_{0}t_{p}\left\{l_{p}-l_{1}\left(1-\cos\theta \right)\right\}}{g-l_{1}\sin\theta } \end{array}$$

In Equation (5), $\varepsilon_{0}$ is vacuum permittivity, 8.85 pF/m, and $t_{p}$ is the thickness of the proof mass or the thickness of the silicon device layer, 30 μm. Notice that capacitances are nonlinear functions of modal variables r and θ. Since we bias the device layer or the proof mass with a DC voltage of $V_{dc}$ and apply the AC voltage of $V_{ac}\cos\left(\omega_{e}t\right)$ to the drive electrode, $V_{XE}$ and $V_{YE}$ are given by,

(6) $$\begin{array}{c} V_{YE}=V_{dc}+V_{ac}\cos\left(\omega_{e}t\right)\\ V_{XE}=V_{dc} \end{array}$$

Further, the constants $\eta_{i}$ and $\alpha_{i}$ in (3) and (4), resulting from Taylor series expansion of capacitances in (5) up to third order, are given by,

(7) $$\begin{array}{c} \eta_{1}=\frac{\varepsilon_{0}t_{p}l_{p}l_{1}}{2g^{2}}\\ \eta_{2}=\frac{\varepsilon_{0}t_{p}l_{1}\left(2l_{p}l_{1}-g^{2}\right)}{2g^{3}}\\ \eta_{3}=\frac{\varepsilon_{0}t_{p}l_{1}\left(6l_{1}^{2}l_{p}-3l_{1}g^{2}-l_{p}g^{2}\right)}{4g^{4}}\\ \;\;\;\;\;\;\;\;\;\;\;\;\;\;\;\;\;\;\;\;\;\;\alpha_{1}=\frac{\varepsilon_{0}t_{p}w_{p}}{2g^{2}}\\ \;\;\;\;\;\;\;\;\;\;\;\;\;\;\;\;\;\;\;\;\;\;\alpha_{2}=\frac{\varepsilon_{0}t_{p}w_{p}}{g^{3}}\\ \;\;\;\;\;\;\;\;\;\;\;\;\;\;\;\;\;\;\;\;\;\;\alpha_{3}=\frac{3\varepsilon_{0}t_{p}w_{p}}{2g^{4}} \end{array}$$

Next, we discuss the characteristics of motion equations given in (3) and (4). Equations (3) and (4) describe a system of two second-order coupled nonlinear ordinary differential equations. The only kind of linear coupling between two modes is the Coriolis coupling due to external angular rate, $\Omega_{z}$. Further, there are two kinds of nonlinearities, inertial and elastic. The nonlinear terms containing the time derivatives of dependent variables, θ and r, are inertial nonlinearities, and the ones not containing the time derivatives of dependent variables are elastic nonlinearities, as referred to in [6]. In addition, when $\Omega_{z}=0$, the Equations (3) and (4) are coupled via only nonlinear terms.

Equations (3) and (4) also estimate the pull-in voltage expressions for both modes. For example, the DC component of $V_{XE}^{2}\alpha_{2}r$ is $V_{dc}^{2}\alpha_{2}r$, which causes electrostatic spring softening by changing the equivalent spring constant of the r mode and thus changing its modal natural frequency, $\omega_{r}$, as well. This term, $V_{dc}^{2}\alpha_{2}r$, when moved to the left hand side of Equation (4) and then using Equation (7) yields the net spring force as

(8) $$F_{net}=\left(K_{r}-V_{dc}^{2}\frac{\varepsilon_{0}t_{p}w_{p}}{g^{3}}\right)r$$

It is known that at the pull-in voltage, $V_{PI,r}$, the gap becomes $g_{PI}=\frac{2}{3}g$ and causes $F_{net}=0$. Substituting this condition for pull-in in (8) yields,

(9) $$K_{r}-V_{PI,r}^{2}\frac{\varepsilon_{0}t_{p}w_{p}}{g_{PI}^{3}}=0$$

At pull-in, the equilibrium between the spring force due to the beams’ stiffness and the electrostatic force in (9) is lost, and the resonating proof mass snaps on the X electrode and the sensor is shorted. Using (9), the pull-in voltage for r mode, $V_{PI,r}$, can be given as follows,

(10) $$V_{PI,r}=\sqrt{\frac{8K_{r}g^{3}}{27\varepsilon_{0}t_{p}w_{p}}}$$

Notice that (10) is the same as the pull-in voltage equation in the literature, e.g., in [51]. Similarly, the pull-in voltage for θ mode, $V_{PI,\theta }$, changes the natural frequency of mode θ, $\omega_{\theta }$, and it can be given as follows,

(11) $$V_{PI,\theta }=\sqrt{\frac{24K_{\theta }g^{3}}{9\varepsilon_{0}t_{p}l_{1}\left(9l_{p}l_{1}-2g^{2}\right)}}$$

Notice in Equation (4), for non-zero angular rate input, $\Omega_{z}$, the term $2M\Omega_{z}r\dot{\theta }$ (quadratic coupled) causes resonance in the sense mode. Since this term is linear in $\Omega_{z}$, the sense mode of the RARS experiences angular rate sensitivity.

The last two terms of electrostatic force in Equation (3), $V_{YE}^{2}\eta_{2}\theta$ and $V_{YE}^{2}\eta_{3}\theta^{2}$, are parametric excitation terms and $V_{YE}^{2}\eta_{1}$ is an explicit time dependent term causing Equations (3) and (4) to be a nonautonomous system for RARS. Moreover, mode r is a non-externally excited mode because Equation (4) has no explicit time dependent terms. In fact, the only way mode r could be excited is through coupling terms, linear and nonlinear. The terms on the right-hand side of Equations (3) and (4) are labelled underneath based on the order of nonlinearity and are self-explanatory.

Lastly, notice that in (4), the non-zero $\Omega_{z}$, makes the centrifugal term, $Mr\Omega_{z}^{2}$, non-zero, and thus decreases, via spring softening, the natural frequency, $\omega_{r}$, of the mode r. However, in real-life applications $\Omega_{z}\ll \omega_{r}$. For example, an input rate of $\Omega_{z}=360^{\circ }/s$ has an ordinary frequency of 1 Hz whereas a typical gyroscope’s sense mode natural frequency is in the order of kHz. This could still limit the frequency bandwidth performance of a conventional mode-matched gyroscope, especially for input rates greater than a couple of Hz. However, unlike a conventional mode-matched MVG that has a pointed frequency response with a narrow frequency bandwidth, the RARS has a flatter and wider (bandwidth) nonlinear frequency response curve and, therefore $\Omega_{z}$ could safely be assumed to have negligible effects on the shifting of $\omega_{r}$.

## 4. Characterization of Sensor’s Nonlinear Dynamics

Although RARS operates at 2:1 AR instead of linear resonance, its Q factor characterization is essential for several reasons. Firstly, the operation of RARS requires a linear resonance, or primary resonance, of its drive mode. The sense mode response at 2:1 AR is directly proportional to the drive mode amplitude. Secondly, the drive mode Q factor, $Q_{d}$, also indicates the mode’s frequency bandwidth at its linear resonance, $\Delta f_{d,LR}$, by $Q_{d}=f_{d}/\Delta f_{d,LR}$ where $f_{d}$ is the linear resonant frequency of the drive mode. There is a trade-off between $Q_{d}$ and $\Delta f_{d,LR}$. Since the sense mode of RARS is designed only to be excited via 2:1 AR, its frequency bandwidth is not bounded by the sense mode’s Q factor, $Q_{s}$. Finally, the modal damping coefficients could be estimated by characterizing RARS for its Q factors and linear resonant frequencies.

**Figure 7:**
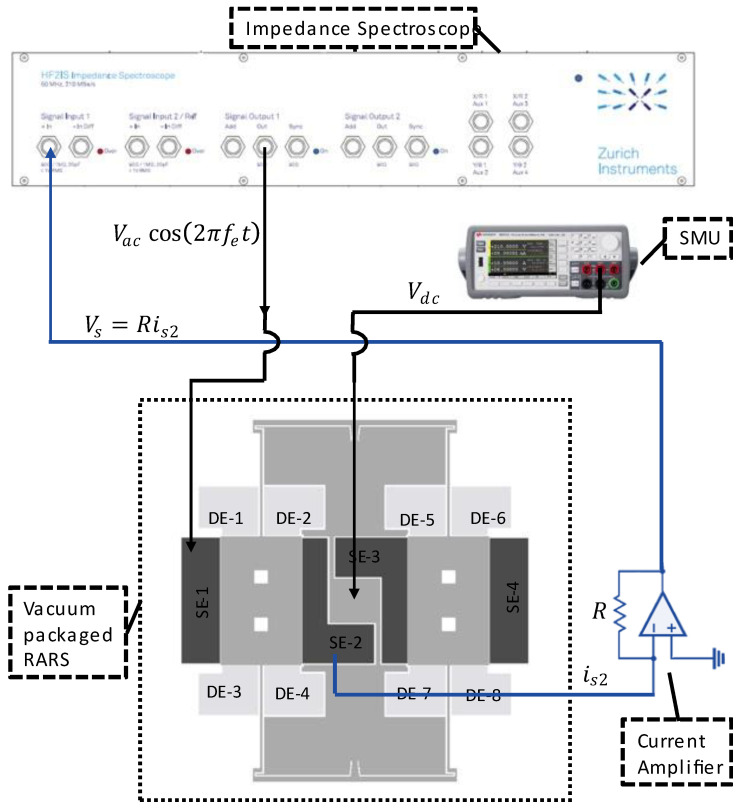
Experimental schematic for Q factor and natural frequency characterization of the sense mode of RARS.

The experimental schematic for the Q factor and natural frequency characterization of the sense mode of RARS is shown in Figure 7. The resonator is biased with DC voltage, $V_{dc}$, applied at the anchor via a source measurement unit or SMU (Keysight B2901A). An AC drive voltage of $V_{ac}\cos\left(2\pi f_{e}t\right)$ is applied to the sense electrode SE-1 via the impedance spectroscope (HF2IS by Zurich Instruments). The drive voltage frequency, $f_{e}$, is swept around the simulated resonant frequency of the sense mode, and the resulting proof mass motion is sensed via the parallel plate capacitance change between the moving proof mass and the stationary electrode, SE-2. Notice that SE-2 detects the small motional current, $i_{s2}$, which is directly proportional to the capacitance change. A transimpedance amplifier or a current amplifier (Femto DHPCA-100) with a variable gain (R) then converts and amplifies the motional current to a detectable voltage signal, $V_{s}=Ri_{s2}$. $V_{s}$ is then analyzed by HF2IS and displayed on a computer via ZI control software. The recorded $Q_{s}$ and the linear resonant frequency of the sense mode, $f_{s}$ are listed in Table 3.

**Figure 8:**
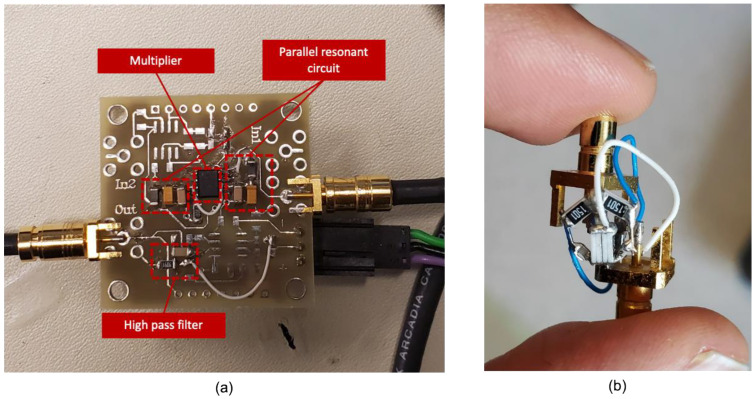
(**a**) Frequency doubler and (**b**) notch filter.

For the 2:1 AR characterization of RARS, we mount the sensor on PCB and use a lock-in amplifier by Zurich Instruments (either HF2IS or HF2LI) along with its ziControl software for signal processing. The 2:1 AR characterization is done with the application of DC bias voltage, $V_{dc}$, to the device and an AC signal, $V_{ac}\cos\left(2\pi f_{d}t\right)$ at the drive mode resonant frequency, $f_{d}$, to the drive electrodes. The sensor’s response in the orthogonal sense mode is differentially sensed at the sense mode resonant frequency, $f_{s}\approx f_{d}/2$, via the sense electrodes. Since in HF2LI we are only able to track a signal at its output excitation frequency, we set the output AC excitation signal in HF2LI to $V_{ac}\cos\left(2\pi f_{s}t\right)$. This would allow us to track the frequency response of the signal in real-time at the sense mode resonant frequency $f_{s}$. However, to realize the primary resonance of the drive mode, we must excite the drive electrodes at $V_{ac}\cos\left(2\pi f_{d}t\right)$ and, therefore, we employ an analog frequency multiplier or simply a frequency doubler circuit, as shown in Figure 8, that takes $V_{ac}\cos\left(2\pi f_{s}t\right)$ as an input signal and outputs $V_{ac}\cos\left(2\pi f_{d}t\right)$ with a reasonable attenuation of the output amplitude. The frequency multiplier circuit takes an input signal and squares it using the parallel resonant LC circuits, made up of surface mount chip inductors, capacitors, and an NPN transistor. The squaring results in the signal with the frequency components at DC and at the first and second harmonics. The signal is then high pass filtered via a passive high pass filter built using a surface mount chip resistor and capacitor to attenuate the first harmonic frequency and the DC component considerably.

**Figure 9:**
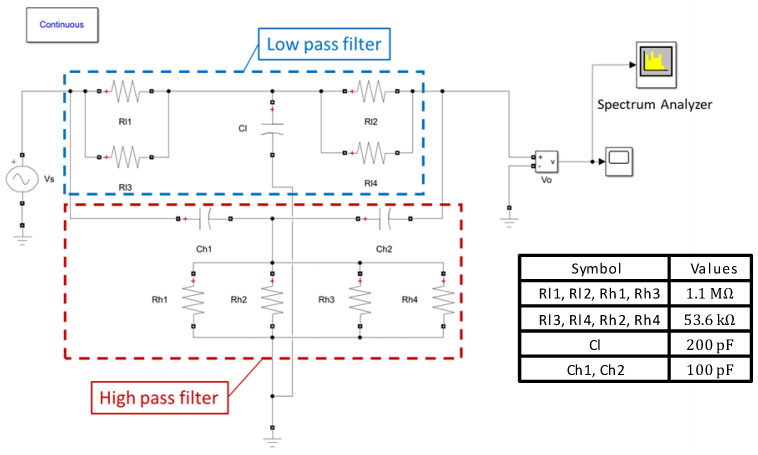
Notch filter circuit.

Further, for specific values of $V_{ac}$, it will be shown in the subsequent section that the sense mode signal due to 2:1 AR exhibits a parasitic feedthrough component at frequency $f_{e}$ that may have a higher amplitude than the component at frequency $f_{s}$. However, in RARS, we set $f_{e}=2f_{s}$, which causes parasitic feedthrough at $2f_{s}$ while the 2:1 AR occurs at $f_{s}$. Although this eliminates the problem of parasitic feedthrough, it is still advisable to attenuate it. Therefore, for the RARS device, we develop a notch filter to attenuate the parasitic feedthrough at $f_{e}\approx 2f_{s}$ from the sense signal. The picture of the notch filter for RARS is shown in Figure 8. Figure 9 shows the circuit, modelled in Simulink, of a passive twin-T notch filter. The upper part shown in the blue rectangle forms a low pass filter, and the lower part in the red rectangle forms a high pass filter. A parallel resistor circuit is chosen because it is easier to solder-stack SMD resistors than to solder them in series. The values of resistors and capacitors are listed in the table as shown in Figure 9. The maximum attenuation is achieved at the notch frequency, $f_{N}=1/\left(4\pi R_{eq}C_{eq}\right)$, where $R_{eq}=R_{h1}\left|\left|R_{h2}\right|\right|R_{h3}||R_{h4}$ and $C_{eq}=C_{h1}$.

**Figure 10:**
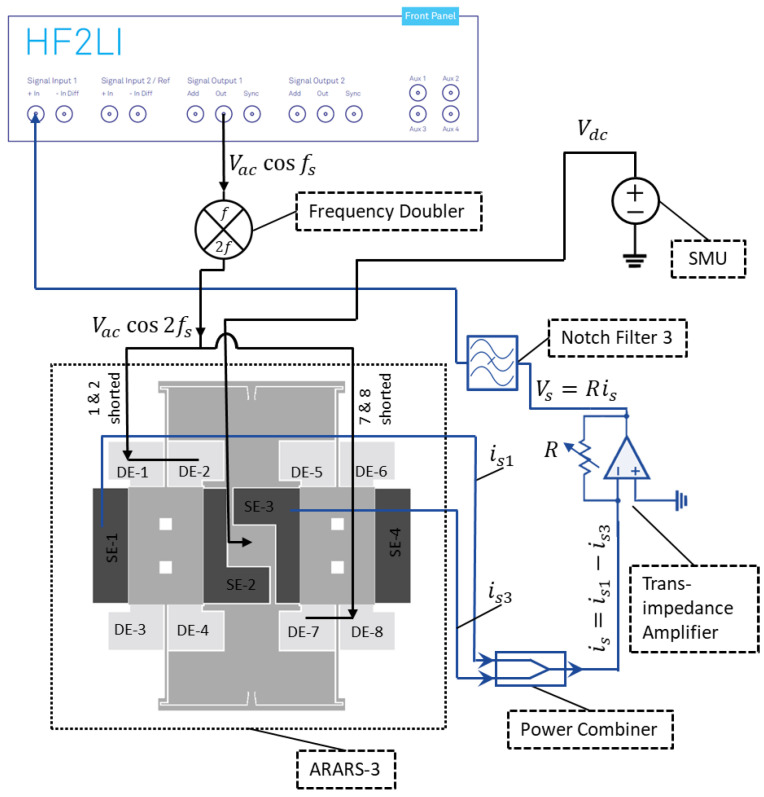
Setup’s schematic for frequency response curves of RARS.

We now characterize the RARS for its 2:1 AR using the experimental setup shown in Figure 10. We apply the DC bias voltage to the device at the anchor via an SMU. As explained earlier, the AC voltage is applied using the frequency doubler to the drive electrode pairs of DE-1 & DE-2 and DE-7 & DE-8. Notice that these pairs consist of two shorted electrodes to maximize the electrostatic force to the proof mass and to optimally utilize the number of available electrical pads for wire bonding on the chip. The sense mode motions of the proof masses are differentially sensed using the sense electrodes SE-1 and SE-3. The notch filter attenuated the amplitude of parasitic feedthrough from the sense signal before feeding it to HF2LI.

**Figure 11:**
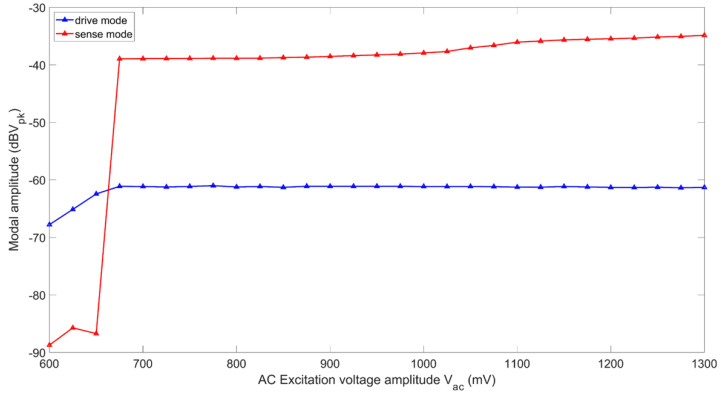
Experimental force response curves of RARS.

Next, we set the input parameters to $V_{dc}=12\;V$, R=10 MV/A, and $f_{e}\approx 2f_{s}$ while varying $V_{ac}$, to obtain the force response curves shown in the semi-log plot of Figure 11. At $V_{ac}=675{\;\mathrm{mV}}_{\mathrm{pk}}$, 2:1 AR occurs, and the sense mode response jumps up from the noise floor. For $V_{ac}<675{\;\mathrm{mV}}_{\mathrm{pk}}$, the drive mode amplitude linearly increases, and after 2:1 AR, it saturates. For $V_{ac}>675{\;\mathrm{mV}}_{\mathrm{pk}}$, the sense mode amplitude steadily increases on a log scale, which indicates considerable increment on a linear scale.

**Figure 12:**
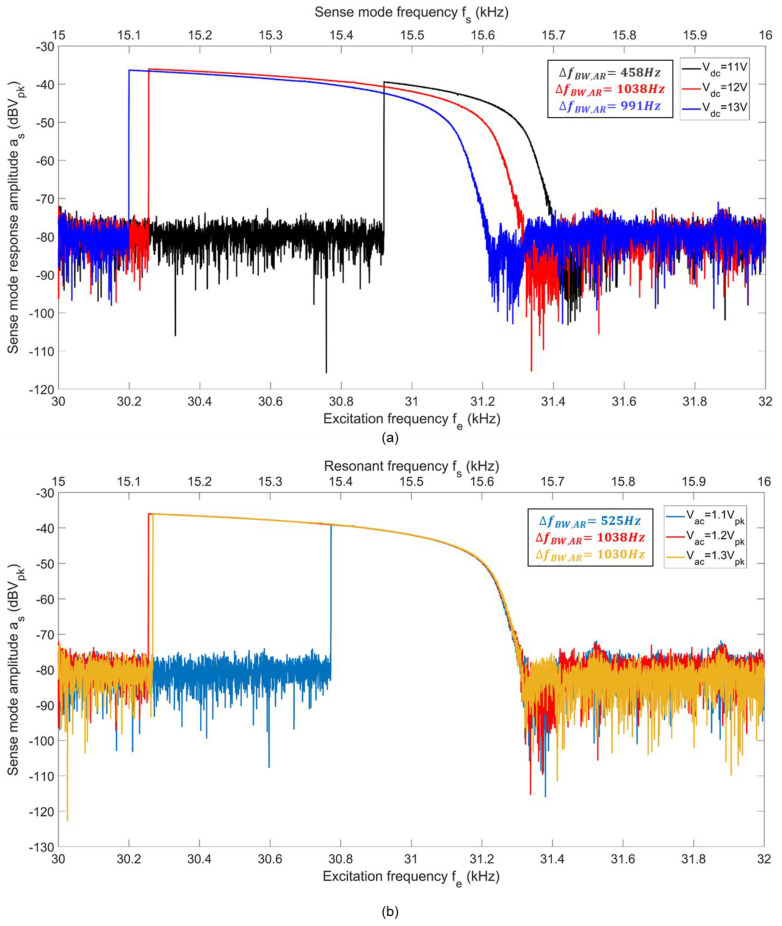
Frequency response curves of the sense mode of RARS at 2:1 AR: while varying (**a**) $V_{dc}$, (**b**) $V_{ac}$.

Finally, to obtain the frequency response curves, we first vary $V_{dc}$ for $V_{ac}=1.2{\;V}_{\mathrm{pk}}$, and then vary $V_{ac}$ for $V_{dc}=12\;V$. Figure 12a,b show frequency responses of the sense mode of RARS for different $V_{dc}$ and $V_{ac}$ values, respectively. For $V_{dc}=12\;V$ and $V_{ac}=1.2{\;V}_{\mathrm{pk}}$, a maximum frequency bandwidth, at 2:1 AR, of $\Delta f_{BW,AR}=1038\;\mathrm{Hz}$ is achieved. Both the figures have two X axes. The bottom axis represents the excitation frequency $f_{e}$ and the top axis indicates the resonant frequency of the sense mode, $f_{s}$.

## 5. Sensor Test Results

**Figure 13:**
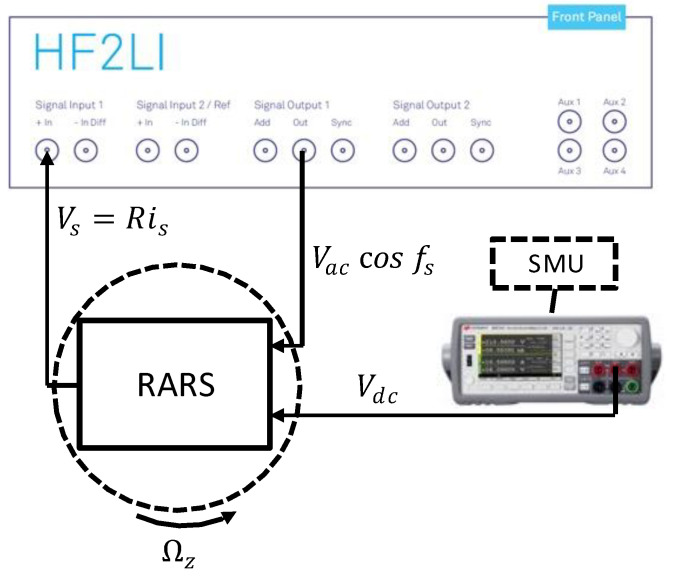
Birds-eye view of the experimental setup for measuring scale factor.

**Figure 14:**
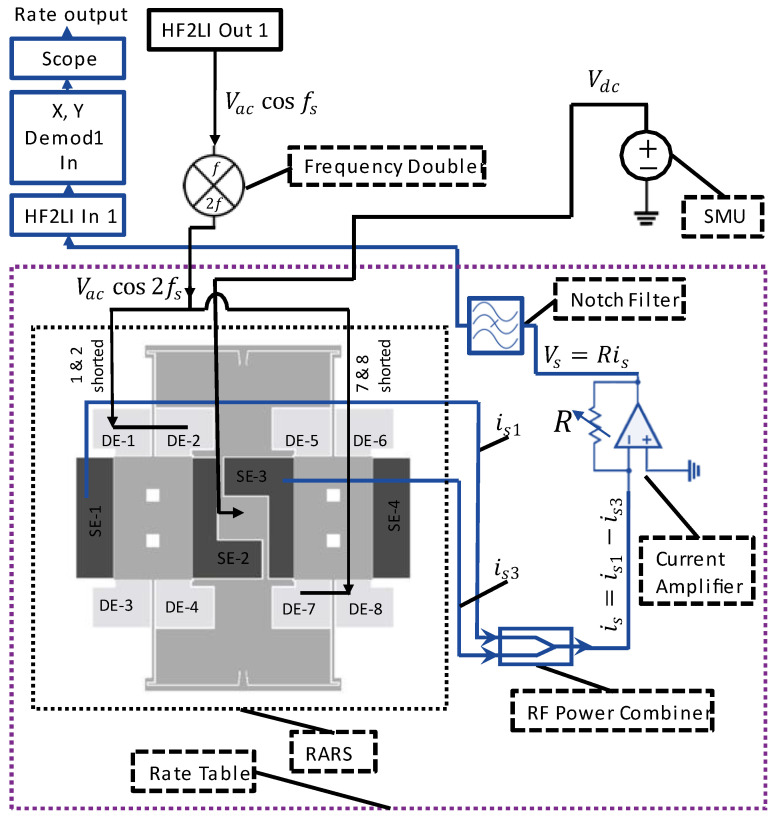
Schematic of the experimental setup for measuring scale factor.

The RARS was tested according to the guidelines of IEEE std 1431 for its scale factor performance [35]. The experimental setup used for these tests is shown in Figure 13 and Figure 14.

Figure 13 shows the schematic of the experimental setup to measure the scale factors of RARS. The required additional circuitry, such as current-to-voltage conversion, is shown in Figure 14. In Figure 13, the sensor-on-PCB is mounted on the base of the rate table (Ideal Aerosmith 1621-200A-TL) with a rate accuracy of ±0.01%, which would subject the sensor to angular rate input of $\Omega_{z}$ with respect to the Z axis, the axis normal to the plane of the sensor. The sensor is biased with the DC voltage $V_{dc}$ supplied by a precision SMU (Keysight B2901A). An AC voltage signal is first fed to the sensor’s two drive electrodes from HF2LI’s signal output port, Out 1, to excite the sensor in anti-phase drive mode. The detected sense mode signal is then fed back to the signal input port, In 1, of HF2LI, which outputs the signal visually on a scope via ZI control software on a computer.

Now, we discuss the detailed experimental setup as shown in Figure 14. Notice that the figure’s blue and black connection lines represent the sense-mode readout circuit and the sensor actuation circuit, respectively. All components inside the purple rectangle of the rate table chamber are securely mounted on the rate table’s rotating base using adhesives and screws. Further, the connection lines crossing the rate table chamber block in the figure indicate wires entering the rate chamber through a slip ring. HF2LI feeds an AC voltage of $V_{ac}\cos\left(2\pi f_{s}t\right)$ the frequency doubler which converts the signal to $V_{ac}\cos\left(2\pi (2f_{s})t\right)$ and feeds to the drive electrodes to resonate the proof mass in anti-phase drive mode.

**Figure 15:**
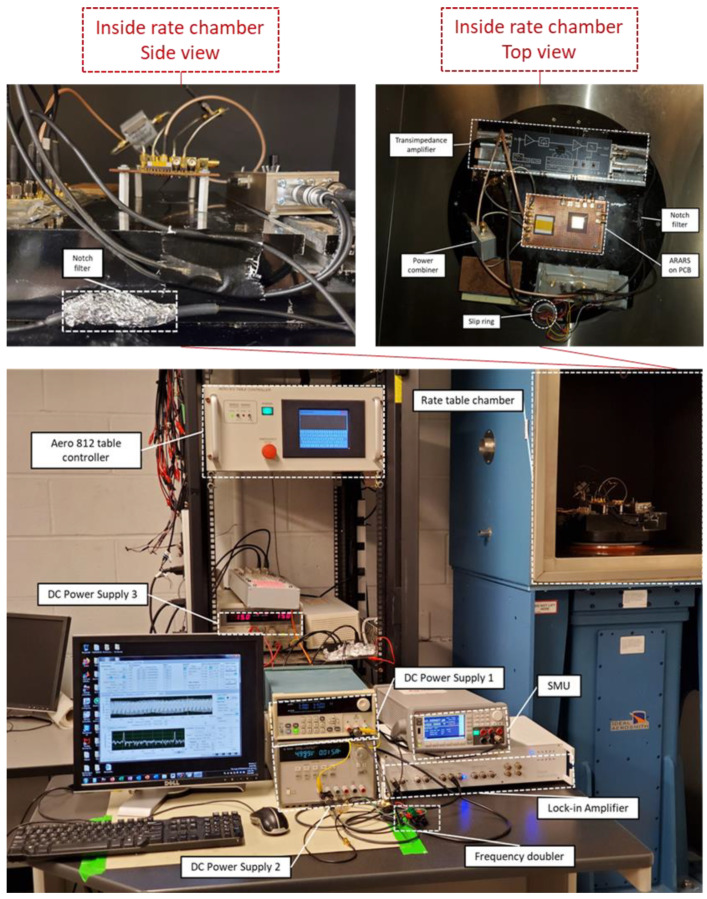
Experimental setup for measuring scale factor.

Figure 15 shows the pictorial description of the experimental setup demonstrated in Figure 14. The RARS is first driven to a stable 2:1 AR, and then a constant angular rate $\Omega_{z}$, a step input, for about 30 s is applied to the rate table via the control panel of the Aero 812 table controller. The filtered sense signal then carries the angular rate information at $f_{s}$ and is measured by HF2LI via the demodulator 1. For each step input, the sensor’s response is recorded via the spectroscope tab of HF2LI. Further, to precisely record the sensor’s output due to angular rate signal at $f_{s}$, we utilize the inbuilt low pass RC filter of HF2LI at a bandwidth of 1 Hz. This allows us to reduce the noise in signal due to the applied angular rate at the sense mode frequency $f_{s}$.

The gyro scale factor, as mentioned in the IEEE std 1431 [35], in mV/°/s for the sensor is then computed by computing the slope of the straight line that can be fitted using the method of least squares from the input-output (°/s−mV) data of the sensor. The input rate limits that confirm the sensor’s computed scale factor with a specific full-scale linearity error, also known as the nonlinearity of scale factor, are also calculated for each sensor. In addition, we calculate the asymmetry error of the scale factor, which is the ratio of the difference in magnitudes of scale factor measured for positive and negative input rates to one-half the sum of the magnitudes. Finally, to test the sensitivity of the scale factor due to variations in operating voltage frequency, we test each sensor’s scale factor at three distinct excitation frequencies. Notice that this last test demonstrates the robustness of RARS over mode-matched MVGs, variations in operating voltage frequency or shift in modal resonant frequencies due to electrostatic softening, aging of the sensor, fabrication imperfections, or applied angular rate.

**Figure 16:**
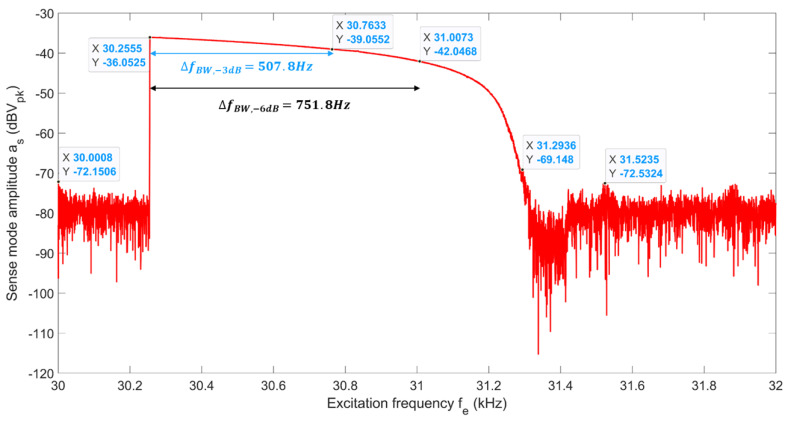
RARS sense mode response to frequency sweep at 2:1 AR.

To test the sensitivity of the scale factor to variations in operating frequency, $f_{e}$, we choose three distinct excitation frequencies. We obtain the scale factors of RARS at its (i) peak resonant frequency,$\;i.e.,\;f_{e}=f_{peak}=30.255\;\mathrm{kHz}$, (ii) the half resonance power (i.e., 29.3% or −3 dB of amplitude drop from its peak) frequency, i.e., $\;f_{e}=f_{-3\mathrm{dB}}=30.763\;\mathrm{kHz}$ and (iii) the quarter resonance power (i.e., 50% or −6 dB of amplitude drop from its peak) frequency, $i.e.,\;f_{e}=f_{-6\mathrm{dB}}=31.007\;\mathrm{kHz}$ of the sense mode’s 2:1 AR response, as shown in Figure 16. The frequency shifts at the half and quarter resonance powers are 1.7% and 2.5% of the frequency at its peak resonance power. Notice that the frequency span on the horizontal axis of Figure 16 is 2 kHz.

**Figure 17:**
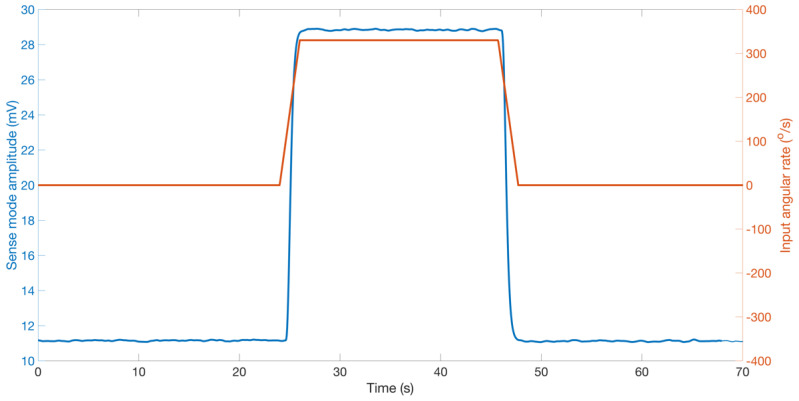
RARS output at $f_{e}=f_{peak}$ to a step input rate of 330°/s.

**Figure 18:**
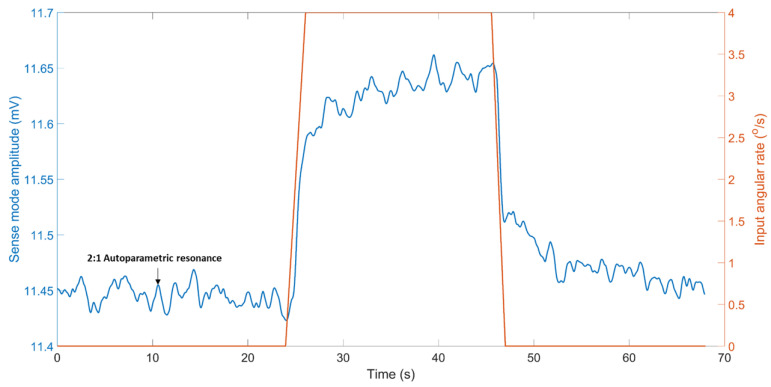
RARS output at $f_{e}=f_{peak}$ to a step input rate of +4°/s.

Next, we test the sensitivity of RARS to angular rate inputs in the range of $\Omega_{z}=\left[\begin{array}{cc} -360 & 360 \end{array}\right]{}^{\circ}/s$ with an interval of 30°/s, and angular acceleration of $\Omega_{z}{}^{\circ}/s^{2}$ for 1 s. Figure 17 demonstrates the sensor’s output at 330°/s. The rise and settling time conform with the input angular acceleration for 1 s. Further, Figure 18 shows the application of RARS in detecting lower angular rates than 5°/s.

**Figure 19:**
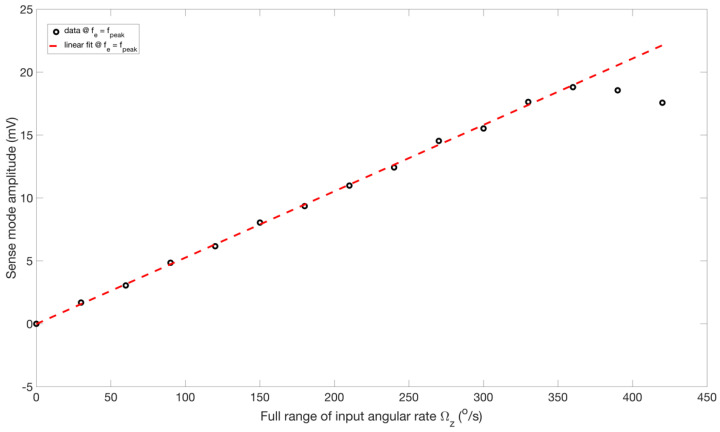
Linear fit for RARS sensitivity measurement.

By using the nominal scale factor value, at $f_{e}=f_{peak}$, Figure 18 predicts the angular rate with an accuracy of 4.06% at the peak response amplitude. Figure 19 demonstrates the comparison of the linear fit of the sensor’s output to the full range of the positive input rates (PIR) at $f_{e}=f_{peak}$. It can be observed that the output of RARS for input rates up to 360°/s are better fitted with a line. For input rates ≥360°/s, however, the input-output relationship becomes more nonlinear.

**Figure 20:**
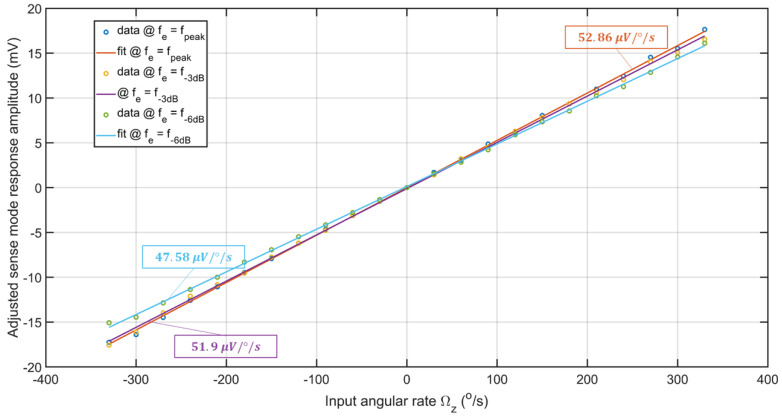
RARS angular rate sensitivity curve with the direction of rotation determined by an external accelerometer.

As shown in Figure 20, for the input rate range of $\Omega_{z}=\left[\begin{array}{cc} -330 & 330 \end{array}\right]{}^{\circ}/s$ the sensor’s output could be fitted with a line using the least squares method. It should be noted that the direction of rotation information is incorporated using an external accelerometer. The scale factors of the sensor for PIR and NIR at $f_{e}=f_{peak}$ are 52.75 μV/°/s and −53.49 μV/°/s, respectively, with an asymmetry error of 1.39%. At $f_{e}=f_{-3\mathrm{dB}}$ for PIR and NIR the scale factors drop by 4.45% and 1.25%, respectively, from those at $f_{e}=f_{peak}$ with an asymmetry error between them of 4.69%. Lastly, at $f_{e}=f_{-6\mathrm{dB}}$ the asymmetry error in scale factors between PIR and NIR is 2.11%, with drops of 8.53% and 11.68% in their respective scale factors from those at $f_{e}=f_{peak}$. Therefore, RARS has better control over the asymmetry errors across all three excitation frequencies. Further, RARS has a full-scale range of $\left[\begin{array}{cc} -330 & 330 \end{array}\right]{}^{\circ}/s$ at all three excitation frequencies. Nominal scale factors of RARS at three distinct excitation frequencies are computed. The drops in nominal factors at $f_{e}=f_{-3\mathrm{dB}}$, and $f_{e}=f_{-6\mathrm{dB}}$ from that at $f_{e}=f_{peak}$ are 1.82% and 10% which indicates that in practice, RARS could handle the frequency shift, shift between its resonance and excitation frequencies, of 1.06% within a total accuracy of 1.82% of its nominal scale factor. The linearity errors in these nominal scale factors approach ±5.5°/s, ±5.5°/s, and ±6.5°/s at $f_{peak}$, $f_{-3\mathrm{dB}}$, and $f_{-6\mathrm{dB}}$, respectively. The full-scale linearity errors of RARS are 0.91% at $f_{peak}$, and $f_{-3\mathrm{dB}}$, and 1.06% at $f_{-6\mathrm{dB}}$. The scale factor and full scale range of the sensor developed by Sarrafan [18] were 11 μv/°/s and ±200°/s, respectively, whereas the RARS demonstrated the maximum scale factor of 52.75 μv/°/s for a full-scale range of ±330°/s.

RARS demonstrated a stable sensitivity of its scale factor to the sensor’s variations in operating frequency with better dynamic range or the input rate limits. In addition, the asymmetry errors are well controlled while operating the sensor at full and half 2:1 AR power. Overall, the sensor demonstrates a limited sensitivity of its output to the frequency mismatch within the half 2:1 AR power.

## 6. Conclusions

This paper presents a single-axis angular rate sensor that is robust to variations in its operating voltage and frequencies, hence dubbed robust angular rate sensor (RARS). Its architecture is designed to operate the sense mode at 2:1 AR to stabilize its scale factor over a wider frequency band by utilizing inertial, damping, and excitation nonlinearities. The sensor is electrostatically actuated in anti-phase drive mode and capacitively sensed using differential sensing via the variable gap capacitive detection.

The in-plane tuning fork design of the sensor allows the induced Coriolis forces experienced by either proof mass to be added to the sense mode response while the common-mode inputs in the same direction are cancelled out. The employed fishhook flexures offer high stiffness in undesired modes while keeping the sensor compliant in drive and sense modes. The sensor structure was modelled analytically and numerically. Pull-in voltage values were calculated to avoid operating the sensor above that voltage. To attain high Q factors, the sensor was fabricated by the wafer-level vacuum encapsulated MIDIS process of Teledyne Dalsa.

Before the angular rate performance tests, the experimental nonlinear characterization of the sensor was carried out. A custom-built notch filter and a frequency doubler circuit were used to attenuate the parasitic feedthrough from the sense signal and provide an AC voltage excitation at the drive mode resonant frequency. The frequency bandwidth, due to 2:1 AR, of 1038 Hz was achieved for the sense mode of RARS. The angular rate performance tests demonstrated that the maximum sensitivity of 52.86 μV/°/s was achieved with a full-scale nonlinearity of 0.91%. The RARS has a dynamic range of ±330°/s, and −3 dB bandwidth of 507.8 Hz.

## Figures and Tables

**Table 1:** Performance summary of MVGs.

Ref.	MVG Type	Sense Axis/Axes	Scale Factor	Full-Scale (°/s)	Nonlinearity (ppm of Full-Scale)	Bandwidth (Hz)	$\mathrm{Noise}\;\mathrm{Density}\;({}^{\circ}/s/\sqrt{Hz})$	$\mathrm{Angle}\;\mathrm{Random}\;\mathrm{Walk}\;({}^{\circ}/\sqrt{h})$	Bias instability (°/h)
[38]	Mode matched	Z	88 mv/°/s	±50	50 k	0.5–10	<1	–	0.32
[39]	Mode matched	X	22 mv/°/s	±200	21.9 k	–	0.02	–	–
[40]	Mode matched	Z	80 μv/°/s	±60	–	4	0.37	6.67	95
[41]	Mode matched	Z	88 mv/°/s	±10	–	1–10	0.0023 ^1^	0.003	0.16
[42]	Mode matched	Z	15.4 mv/°/s	±50	–	42	0.0007 ^2^	0.036	1.6
[43]	Mode matched	X, Y, Z	–	±100	500	80	0.0039	0.23	1.2
[44]	Mode matched	Z	3.79 mv/°/s	±100	28	15	–	4.96	39.54
[45]	Mode split	X, Y	1.71 mv/°/s	±300	3.1 k	160	0.012	–	72
[46]	Mode split digital	Z	47.3 LSB/°/s	±500	770	120	0.0075 ^2^	0.45	9.6
[47]	Mode split	Z	2 mv/°/s	±300	1.8 k	95	0.25 ^1^	0.008	0.08
[48]	Mode split	Z	9.29 mv/°/s	–	59.3	104	–	0.0416	0.445
[18]	AR-based	Z	11 μv/°/s	±200	–	–	–	–	–

^1^ Actual values are given in $\mathrm{nV}/\sqrt{\mathrm{Hz}}$ but using corresponding scale factor the values are converted to ${}^{\circ}/s/\sqrt{\mathrm{Hz}}.\;$^2^ Actual values are given in ${}^{\circ}/h/\sqrt{\mathrm{Hz}}$.

**Table 2:** Dimensions and properties of the RARS.

Parameter	Value	Parameter	Value
$l_{p}$	240 μm	$b_{4}$	$300\times 16{\;µm}^{2}$
$w_{p}$	380 μm	$t_{p}$	30 μm
$b_{1}$	$313\times 7{\;µm}^{2}$	$\rho_{Si}$	$2330\;\mathrm{kg}/m^{3}$
$b_{2}$	$28\times 11{\;µm}^{2}$	g	1.75 μm
$b_{3}$	$40\times 5{\;µm}^{2}$	$\varepsilon_{0}$	8.854 pF/m
$l_{v}$	40 μm	$w_{v}$	40 μm

**Table 3:** Q factor and resonant frequency characterization of RARS.

Parameter Type	Parameter	Values
Input	DC bias voltage, $V_{dc}\left(V\right)$	12
	AC voltage amplitude, $V_{ac}\left(mV_{pk}\right)$	150
	Amplifier gain, R(kV/A)	100
Output	Drive mode resonant frequency, $f_{d}\left(\mathrm{kHz}\right)$	30.607
	Sense mode resonant frequency, $f_{s}\left(\mathrm{kHz}\right)$	15.197
	Drive mode Q factor, $Q_{d}$	13,780
	Sense mode Q factor, $Q_{s}$	12,920

## Data Availability

Not applicable.
